# Selenium as a Modulator of Redox Reactions in the Prevention and Treatment of Cardiovascular Diseases

**DOI:** 10.3390/antiox13060688

**Published:** 2024-06-03

**Authors:** Klaudia Leszto, Laura Biskup, Klaudia Korona, Weronika Marcinkowska, Maria Możdżan, Andrzej Węgiel, Ewelina Młynarska, Jacek Rysz, Beata Franczyk

**Affiliations:** 1 Department of Nephrocardiology, Medical University of Lodz, Ul. Zeromskiego 113, 90-549 Lodz, Poland; claudial@op.pl (K.L.);; 2 Department of Nephrology, Hypertension and Family Medicine, Medical University of Lodz, Ul. Zeromskiego 113, 90-549 Lodz, Poland

**Keywords:** selenium, antioxidant, oxidative stress, cardiovascular diseases

## Abstract

Cardiovascular diseases stand as the predominant global cause of mortality, exerting a profound impact on both life expectancy and its quality. Given their immense public health burden, extensive efforts have been dedicated to comprehending the underlying mechanisms and developing strategies for prevention and treatment. Selenium, a crucial participant in redox reactions, emerges as a notable factor in maintaining myocardial cell homeostasis and influencing the progression of cardiovascular disorders. Some disorders, such as Keshan disease, are directly linked with its environmental deficiency. Nevertheless, the precise extent of its impact on the cardiovascular system remains unclear, marked by contradictory findings in the existing literature. High selenium levels have been associated with an increased risk of developing hypertension, while lower concentrations have been linked to heart failure and atrial fibrillation. Although some trials have shown its potential effectiveness in specific groups of patients, large cohort supplementation attempts have generally yielded unsatisfactory outcomes. Consequently, there persists a significant need for further research aimed at delineating specific patient cohorts and groups of diseases that would benefit from selenium supplementation.

## 1. Introduction

Cardiovascular disease (CVD) is the primary cause of global mortality and significantly contributes to a decline in quality of life [1,2,3]. Based on statistical data, in 2019, the mortality attributed to CVD was 17.8 million, and projections suggest that by 2030, this figure is expected to increase to 23 million [2,3]. In order to reduce the prevalence of CVD within the population, it is important to take into consideration not only the spectrum of CVD risk factors but also the underlying pathomechanisms governing the progression of these disorders [4]. Increased oxidative stress is recognized as an important contributor to CVD pathogenesis, precipitating conditions including myocardial infarction, ischemia or reperfusion injury, and heart failure [5]. The excessive generation of reactive oxygen species (ROS) leads to a reduction in nitric oxide bioavailability, thereby promoting vasoconstriction and eliciting arterial hypertension. Additionally, ROS disrupt calcium homeostasis in the myocardium, resulting in arrhythmias and facilitating cardiac remodeling through signaling hypertrophy and apoptosis. Moreover, ROS are key factors in instigating the formation of atherosclerotic plaques [4]. 

In recent years, numerous studies have explored the use of antioxidants as therapeutic agents. One particular trace element, Se, has garnered special attention [6,7]. Se holds a significant and unique role among microelements. It serves as an essential component of the 21st amino acid, selenocysteine (SeC), which is found within the catalytic sites of selenium-dependent enzymes. Proteins containing at least one SeC residue are termed selenoproteins and play numerous crucial physiological roles, primarily centered on maintaining cellular redox balance [8]. 

In addition to its antioxidant properties, Se can affect cell survival through the expression of proapoptotic proteins and regulation of autophagy in myocardial cells. Activation of caspase family genes leads to apoptosis both through the death receptor and through the mitochondrial pathway, in which the antiapoptotic factor BCL-2 is a major regulator [9]. Se deficiency leads to an increase in mRNA levels of caspase-3 and caspase-9 and a decrease in BCL-2 levels, which promotes cardiomyocyte death [10]. In addition, Se deficiency results in reduced expression of potassium channels, STAT3 activity, and mitochondrial function [11]. The STAT/JAK pathway mediates inflammation-related cytokines, regulating growth, survival, and differentiation. Se supplementation by increasing STAT3 protein may play a cardioprotective role after ischemia-reperfusion injury [12,13].

**Figure 1:**
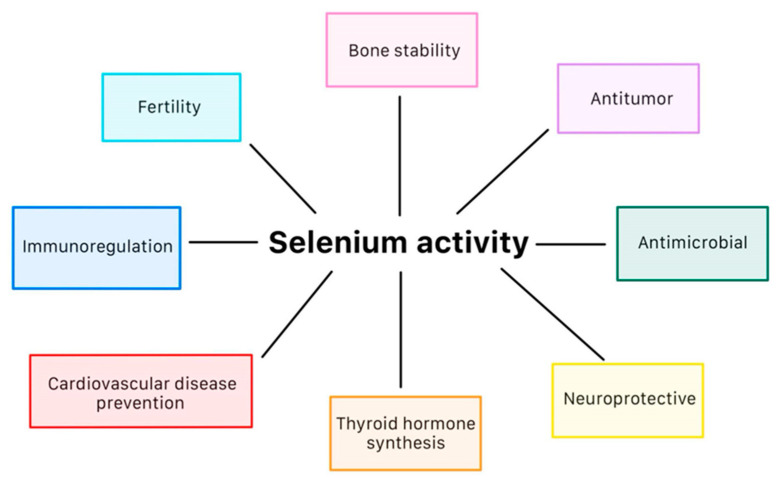
Effects of selenium on human body [61].

A comprehensive collection of 25 selenoprotein genes has been documented, which are distributed throughout various organs and tissues displaying diverse substrate specificity [14]. Among these proteins, there are five glutathione peroxidases, three iodothyronine deiodinases, three thioredoxin reductases, selenophosphate synthetase 2, methionine sulfoxide reductase B1, and selenoproteins F, H, I, K, M, N, O, P, S, T, V, and W [15,16]. The diverse occurrence of selenoproteins throughout the body enables them to potentially modulate pathogenetic mechanisms in numerous disease processes (Table 1, Figure 1) [14]. The significance of heart-specific selenoproteins is underscored by Benstoem et al., who demonstrated that low Se levels and subsequent removal of selenoproteins through deletion of SeC tRNA in the heart and skeletal muscles resulted in sudden cardiac arrest due to increased oxidative stress and inflammation [17]. 

Despite recent advances in CVD therapies, there are still many factors that need to be considered. The effect of dietary supplementation is an understudied topic; however, research suggests that it may influence the progression of CVD. This review illustrates the antioxidant role of Se, shedding light on its influence on the pathomechanisms underlying arterial hypertension, coronary heart disease, heart failure, and arrhythmias.

**Table 1:** Types of selenoproteins in humans and their functions and role in various diseases.

Proteins with Se	Functions	Related Disorders	References
Selenoproteins			
F	Involvement in ER redox protein folding process	Colorectal cancer Gastric cancer	[18]
	Regulation of ER stress		
H	Regulation of redox homeostasis	Gastrointestinal tumor	[19,20]
	Protection against UVB-induced apoptosis		
	Protection against oxidative stress		
	Inhibition of cellular aging		
	Maintenance of genome integrity		
	Suppression of oxidative. DNA damage in tumorigenesis		
I	Involvement in membrane lipid synthesis of phosphatidylethanolamine (PE) and plasmenyl PE	Hereditary spastic paraplegia	[21,22]
	Neuronal development and myelin synthesis		
	Regulation of T-cell function		
K	Degradation of misfolded proteins	Melanoma Gastric cancer Alzheimer’s disease Atherosclerosis	[21,23,24,25]
	Regulation of redox equilibrium		
	Antioxidant activity in cardiomyocytes		
	Modulation of calcium homeostasis in immune cells		
	Regulation of neuronal apoptosis induced by ER stress		
	Palmitoylation		
M	Antioxidant	Alzheimer’s disease Non-alcoholic fatty liver Disease Solid tumors	[26,27]
	Hypothalamic leptin signaling		
	Bone development		
	Neuroprotective		
	Role in body weight and energy metabolism		
	Calcium homeostasis		
N	Oxidative and calcium homeostasis in endoplasmic reticulum	SEPN1-related myopathies	[28,29]
	Muscle regeneration and satellite cell maintenance in skeletal muscle		
O	Proper cellular response to oxidative stress Transfers AMP from ATP to Ser, Thr, and Tyr residues on protein substrates (AMPylation)	Staphyloenterotoxemia Ritter’s disease	[30]
P	Antioxidant	Cancers Ataxia and seizures CVDs	[31,32,33]
	Maintain neuronal activity		
	Regulate pancreatic β cell function		
	Transport Se to tissues		
R	Antioxidant	Cataracts	[34]
	Repairing oxidized proteins		
S	Regulate inflammation	Hashimoto’s Thyroiditis CVDs	[35,36]
	Induce ER stress apoptosis		
	Immune regulation		
T	Antioxidant role in cardiomyocytes	Parkinson’s disease Embryonic lethality in mice	[37,38]
	Protection of the heart against ischemia/reperfusion injury		
	Hormone synthesis		
	Calcium mobilization		
	Neuroprotective		
	Control of glucose homeostasis		
V	Spermatogenesis	Azoospermia	[15,39]
W	Oxidation	Osteoporosis Anemia	[40,41]
	Regulate bone metabolism		
	Support erythroblast and muscle development		
Enzymes			
Iodothyronine deiodinases (DIO)	Act as biocatalysts for the regulation of the activity of thyroid hormones		[42]
DIO1	Participation in the production of the active thyroid hormone T3	Various thyroid-related disorders	[14,42]
	Conversion of T4 to a reduced form T3 (rT3)		
DIO2	Participation in the production of the active thyroid hormone T3	Various thyroid-related disorders	[14]
DIO3	Contributes to the generation of the inactive rT3 and T2	Various thyroid-related disorders	[14,42]
	Conversion of T4 to rT3 via inner-ring deiodination of T4		
Thioredoxin reductases (TrxR)	Involved in cellular antioxidative defense systems and the maintenance of intracellular redox states to maintain cell viability		[14]
TrxR1	Redox regulation	Cancers	[43,44]
	Antioxidant defense		
	Maintenance of intercellular reducing conditions		
TrxR2	Redox signaling	Bone metabolic diseases Embryonic lethality in mice	[45,46]
	Metabolism, proliferation, differentiation, migration, and apoptosis of cells		
RGR (Retinal G Protein Coupled Receptor)	Involved in the visual cycle-regeneration of visual pigments in the retina	Retinitis pigmentosa	[47,48]
	Receptor for all-trans- and 11-cis-retinal		
Glutathione peroxidases (GPx)			
GPx1	Antioxidant	Atherosclerosis Myocardial infarction Alzheimer’s disease Parkinson’s disease Cerebral ischemia Breast cancer Diabetes mellitus type 2	[49,50,51,52,53]
GPx2	Antioxidant	Colorectal cancer Breast cancer Prostate cancer Liver cancer Non-small-cell lung cancer	[54,55]
	Inflammation prevention		
	Mucosal homeostasis		
GPx3	Antioxidant	Colorectal cancer Gastric cancer Thyroid cancer	[56]
GPx4	Antioxidant	Systemic lupus erythematosus Neurodegenerative diseases	[57,58,59]
	Ferroptosis regulator		
	Spermatogenesis		
GPx6	Antioxidant	Unknown	[60]

## 2. The Application of Se in the Prevention and Treatment of CVD

### 2.1. Hypertension

Hypertension stands as a significant global health concern, as it is a leading cause of mortality worldwide, accounting for around 14% of all deaths [62]. The World Health Organization (WHO) estimates that hypertension affects over one billion people globally, with its prevalence steadily increasing over the past few decades [63].

Characterized by persistently elevated blood pressure levels, hypertension is a major risk factor for various adverse health outcomes, including heart disease, stroke, and kidney failure. Dietary factors, including high sodium intake, low potassium intake, excessive alcohol consumption, and diets high in saturated fats and processed foods, have been identified as significant contributors to the development and progression of hypertension [64]. Additionally, trace elements have garnered increasing attention in recent studies as potential contributors to hypertension. Selenoproteins, containing SeC as a prominent Se compound, act as antioxidants and regulators of redox processes, protecting cells from oxidative stress and free radical damage [65]. Immune system functionality, oxidative stress, and inflammatory reactions are involved in the onset and advancement of hypertension [66].

The molecular mechanisms underlying hypertension remain unclear, although inflammation has been implicated in its onset and progression [67]. Studies have shown a significant association between inflammatory markers and hypertension risk, with elevated levels of hs-CRP and IL-6 linked to increased disease incidence [68]. Se supplementation has been found to reduce CRP and IL-6 plasma concentrations by controlling the expression of selenoprotein genes and preventing NF-κB activation [69]. Additionally, Se may enhance nitrogen oxide (NO) synthesis by stimulating NO synthase activity [70] and inhibiting NO degradation, thereby promoting vasodilation [70,71]. Arterial stiffness, which also plays a significant role in hypertension, is associated with vascular remodeling characterized by changes in vessel structure and function [72]. Inflammatory factors, including IL-1, IL-17, and IL-6, are stimulated by Angiotensin II signaling, further contributing to vascular remodeling and arterial stiffness [73]. Anti-inflammatory properties of Se may help to mitigate vascular remodeling, reduce inflammation, and lower the risk of hypertension.

In a study conducted by Bastola et al. in 2020, analyzing data from 6683 participants in the National Health and Nutrition Examination Survey (NHANES) spanning from 2011 to 2016, it was observed that there exists a significant positive correlation between serum Se levels and hypertension, regardless of age or intake of antihypertensive medications [74]. The analysis revealed that serum Se levels at or above 120 μg/L (with a reference range about 70–150 μg/L) were notably associated with hypertension (OR = 1.46, 95% CI = 1.29–1.66) after adjusting for potential confounding variables [74]. Moreover, an elevated serum Se level exceeding 150 μg/L exhibited a strengthened association with hypertension (OR = 1.69, 95% CI = 1.32–2.17) [74]. Furthermore, the results indicated that an increase in serum Se levels was more pronounced in diastolic blood pressure measurements compared to systolic blood pressure measurements [74]. This observation aligns with previous findings reported by Mark et al., where dietary Se supplementation in a nutritionally deficient population resulted in the development of diastolic hypertension without affecting systolic blood pressure [75].

However, the influence of serum Se levels on hypertension risk remains a topic of controversy. Recent research contradicts the findings of Bastola et al. [74], suggesting that elevated Se consumption is associated with a decreased risk of hypertension.

Wu et al.’s research investigates the relationship between dietary Se intake and hypertension, marking a novel addition to previous studies that mainly focused on serum Se levels. Data from NHANES spanning from 2003 to 2018 were utilized to examine the link between dietary Se intake and the occurrence of hypertension in adults. Among 32,928 participants with an average dietary Se intake of 1.12 ± 0.53 μg, the overall hypertension prevalence was 36.55% [76]. Hypertension rates decreased across higher quartiles of dietary Se intake. Each quartile increase in dietary Se intake was associated with an 11% reduced likelihood of hypertension prevalence (OR = 0.89; 95% CI: 0.80–1.00; *p* = 0.0425) [76]. Subgroup analyses indicated no significant correlations between gender, age, body mass index, smoking status, alcohol consumption, diabetes mellitus, and the association between dietary Se intake and hypertension prevalence [76].

As these investigations were epidemiological in nature and relied on self-reported dietary data from surveys, susceptible to subjective bias, they established an association between serum Se levels or Se intake and hypertension, without necessarily establishing a direct cause-and-effect relationship. In contrast, a recent experimental study revealed that rats subjected to a low-selenium diet developed hypertension due to decreased urine sodium excretion [77]. The hypertension observed in Se-deficient rats was linked to elevated renal angiotensin II type 1 receptor (AT1R) expression and activity, which contributed to increased reabsorption of sodium in renal tubules [77]. The decreased Se intake resulted in reduced levels of GPx1, a selenoprotein, which via the NF-κB pathway leads to the up-regulation of AT1R expression [77].

In 2021, a study aimed to investigate the relationship between serum Se levels and mortality in hypertensive patients [78]. Utilizing data from NHANES 2003–2004, participant outcomes were tracked until December 31, 2015. A total of 929 individuals were included in the analysis, with 307 deaths observed, including 56 cardiovascular fatalities, over an average follow-up period of 121.05 ± 40.85 months. A U-shaped association was noted between serum Se levels and both all-cause and cardiovascular mortality. Participants were stratified into quartiles based on serum Se levels (Q1: ≤124 μg/L, Q2: 125–135 μg/L, Q3: 136–147 μg/L, Q4: ≥148 μg/L) [78]. In a fully adjusted model, comparisons among quartiles revealed that individuals in the third quartile (Q3) had lower risks of both all-cause [HR (95% CI), 0.57 (0.39–0.81)] and cardiovascular mortality [HR (95% CI), 0.33 (0.13–0.86)] [78]. The lowest mortality rates for all-cause and cardiovascular deaths were observed at serum Se levels of 136 μg/L and 130 μg/L, respectively [78].

Se therapy holds promise as a potential agent in managing hypertension, a prevalent global health issue associated with significant morbidity and mortality. While epidemiological studies suggest a correlation between serum Se levels and hypertension risk, experimental research in animal models has provided physiological insights into the role of Se in blood pressure regulation. However, the exact nature of the relationship between Se and hypertension requires further investigation, including well-designed clinical trials to assess the efficacy and safety of Se supplementation in hypertensive individuals. A better understanding of selenium’s therapeutic potential in hypertension may offer new strategies for prevention and treatment in clinical practice.

### 2.2. Coronary Heart Disease

Coronary heart disease (CHD) is a common CVD that is one of the leading causes of morbidity and mortality worldwide. Complex interactions between multiple genetic and environmental factors are involved in its pathogenesis [79]. An important factor in the development and progression of CHD is oxidative stress, which contributes to endothelial dysfunction. Consequently, antioxidants, especially those from the diet, have become the focus of several studies for their role in the prevention of CHD.

Se is a trace element that, through the activity of GPx and other selective selenoproteins, prevents lipid peroxidation and oxidative stress and presents anti-inflammatory effects [80]. It has a significant role in both the acute and chronic phases of CHD. Se plays a crucial role in the neutralization of reactive oxygen and nitrogen species, contributing to the reduction in ischemic injury and left ventricular hypertrophy. In the literature, Se deficiency is found in most patients after myocardial infarction (MI) [81,82]. In a clinical study of 84 patients with acute MI, lower levels of Se were observed in plasma, erythrocytes, and nails compared to controls [83]. Furthermore, another study observed in patients with acute MI a significant correlation between baseline Se levels and peak troponin I levels, which is linked to myocardial necrosis, suggesting an association between Se changes and the extent of MI [84]. Venardos et al. revealed in a rat model that the hearts of selenium-deficient animals were more prone to ischemia-reperfusion injury with increased protein and lipid peroxidation [85]. On the other hand, Zachara et al. observed that blood Se levels in patients with MI did not differ compared to the control group and remained stable throughout the study period [86].

The effect of Se on the development of CHD and its association with mortality remains controversial. A meta-analysis by Flores-Mateo et al. revealed a statistically significant inverse association between Se levels and CHD risk in observational studies [87]. They revealed that a 50% increase in Se levels corresponded to a 24% reduction in coronary incident risk. Additionally, this meta-analysis showed that the pooled relative risk of CHD based on a ratio of the highest and lowest Se levels was 0.85 (95% CI, 0.74–0.99). However, the validity of the association in the observational studies analyzed was uncertain [87]. Moreover, Kuria et al. showed that physiologically high levels of Se in the body were related to reduced CHD mortality (RR¼ 0.73, 95% CI: 0.59–0.91), as well as reduced CHD incidence (RR¼ 0.83, 95% CI: 0.74–0.94) [88]. Higher plasma Se concentrations were correlated with lower CHD risk in a nested case–control study in prospective Dongfeng-Tongji cohort [89]. An evaluation of the non-linear relationship of dietary Se intake and serum Se concentration with CHD incidence was conducted by Xie et al. This study included data on 17,867 participants from the United States collected from NHANES between 1999 and 2006. It showed a negative correlation between serum Se concentration and CHD (OR: 0.989, 95% CI: 0.981, 0.997, *p* = 0.006), which was nonlinear after adjusting for multiple variables. Participants with higher serum Se levels had a reduced incidence of CHD, and with further increases in serum Se levels, the incidence of CHD decreased slowly. Furthermore, Xie et al. observed a U-shaped curve representing the relation between dietary Se intake and all-cause mortality. In contrast, there was no statistically significant association between dietary Se intake and the risk of developing CHD (*p* = 0.206) in an analysis including confounders [90].

In other studies, there was no significant correlation between decreased Se levels and increased CHD risk or mortality [91,92,93]. It has been suggested that discrepancies between studies may be due to differences in baseline Se levels in subjects depending on geographic region. The protective effects of Se are observed in regions with low Se levels, while in individuals with sufficient Se levels, supplementation may have no additional benefit. Other reasons for the inconsistent findings include socioeconomic factors, differences in patients’ risk profiles, and other variables affecting Se concentrations [88,93,94,95]. A meta-analysis of 16 randomized controlled trials involving 43,998 participants showed that Se supplementation did not reduce coronary heart disease mortality or affect lipid profile. Nevertheless, it revealed a substantial effect of increased Se supply on the increase in GPx activity (SMD = 0.5; 95% CI, 0.36–0.64; *p* < 0.001) and on the decrease in serum CRP levels (SMD = −0.48; 95% CI, −0.96 to 0; *p* = 0.049), which is one of the risk factors for CHD due to its direct correlation with the presence and severity of coronary atherosclerosis [96]. Similar results on the effect on GPx activity in endothelial cells were obtained by Schnabel et al. based on in vitro cell culture and in vivo clinical trial data [97].

Evidence suggesting an association between blood Se and CHD remains insufficient. Further studies in larger patient cohorts are needed to investigate selenium-dependent effects at the molecular level to provide reliable data on the preventive and diagnostic use of Se in CHD in clinical practice.

### 2.3. Heart Failure

Heart failure (HF) stands as a significant global health concern, marked by persistent challenges in enhancing patient quality of life and long-term survival despite advancements in therapeutic interventions. HF is defined as a functional and structural abnormality of the cardio-vascular system, which results in increased pressure in the heart or a deficient cardiac output during resting or exercising [98]. Within the pursuit of novel strategies for HF prevention and management, attention has turned towards the potential impact of Se.

Se emerges as a pivotal element in both the prevention and treatment paradigms of HF, yet its precise role warrants comprehensive examination. Understanding the significance of Se and its correlation with HF is essential. Fundamental to note are the diverse pathophysiological mechanisms, including oxidative stress, inflammation, and nutrient deficiencies, all contributing to the development and progression of HF.

There are many publications dedicated to the topic of Se deficiency and HF. The study of Mirdamadi et al. showed an association between low level of Se and a high incidence of HF [99]. A total of 32 hospitalized patients with HF and 32 healthy controls were enrolled in the case–control study. The serum Se levels were compared in case and control groups. The mean serum Se was 92.5 ± 22.44 mg/dL in patients with HF and 109.3 ± 29.62 mg/dL in healthy controls (*p* = 0.013). This study showed statistically significant lower levels of serum Se in patients with HF in comparison to healthy individuals. Furthermore, a noteworthy negative correlation was noted between Se levels and both left ventricular volume and pulmonary artery pressure (rho = −0.39, *p*= 0.03 and rho = −0.45, *p* = 0.01, respectively). Additionally, although not statistically significant, a positive trend was noted between the Se level and left ventricular ejection fraction. This observation potentially indicates the impact of Se deficiency on cardiac function and HF pathogenesis [99].

A prospective cohort study by Bomer elucidates the adverse implications of Se deficiency on patient outcomes, with impaired mitochondrial function and oxidative stress underscored as potential underlying mechanisms. The study enrolled 2516 patients with worsening HF to investigate Se deficiency in their serum. Additionally, the investigation dived into potential mechanisms by which Se deficiency might affect prognosis, by culturing human cardiomyocytes without Se and assessing mitochondrial function and oxidative stress. In cultured human cardiomyocytes, Se deprivation impaired mitochondrial function and oxidative phosphorylation, which led to an increase in intracellular ROS levels [100].

The study revealed that over 20% of patients had serum Se concentration under <70 μg/L. Moreover, the Se deficiency was associated with poorer quality of life, exercise capacity, and worse prognosis. Serum concentrations of 70–100 μg/L had similar adverse associations. Notably, supplementation trials exhibit promising amelioration of clinical symptoms in HF patients, hinting at selenium’s therapeutic potential [100]. Recent meta-analysis performed by Jenkins, which used data from 43 different studies (N = 114,146), examined the effect of antioxidant mixtures in relation to mortality. Antioxidant mixtures were defined as a combination of two or more of the following molecules: vitamin A, retinol, β-carotene, vitamin C, vitamin E, Se, zinc, and copper. They concluded that there was a significant reduction in both cardiovascular and all-cause mortality when Se was included in the antioxidant mixture (RR 0.77; 95% CI 0.62, 0.97 and RR 0.90; 95% CI 0.82, 0.98, respectively) [101].

These results were not observed when Se was not part of the antioxidant mixture [101]. However, the existing body of evidence remains insufficient to advocate widespread Se supplementation in HF management, necessitating further high-level research to delineate its precise role and therapeutic efficacy. Prospective cohort studies are warranted to ascertain the causative link between Se deficiency and HF incidence, shedding light on the nuanced interplay between Se and cardiac homeostasis.

In conclusion, Se emerges as a promising adjunct in HF management, holding potential for enhancing patient outcomes. While its precise mechanisms and therapeutic implications necessitate further elucidation, Se supplementation holds promise as a viable strategy for HF intervention, pending rigorous validation through high-level evidence. Efforts to unravel the intricate interplay between Se deficiency and HF pathophysiology remain imperative, paving the way for optimized therapeutic strategies and improved patient care.

### 2.4. Arrhythmias

Atrial fibrillation (AF) is the most common cardiac arrhythmia. Its prevalence varies from 2% to 12%, gradually increasing over age [102]. Numerous contributing factors have been postulated to intricately participate in the pathomechanism of AF, including inflammatory processes, fibrotic remodeling, and heightened oxidative stress [103]. Excessive ROS generation is considered a direct factor affecting propagation of action potential and functioning of the ion channels [104]. As a result of these findings, attempts were made to determine the role of microelements such as Se in AF development and the potential impact of its supplementation in antiarrhythmic treatment.

In the PREVEND study, a total cohort of 6000 patients with urinary albumin excretion ≥10 mg/L and 2592 participants with excretion less than 10 mg/L were qualified for evaluation in 3-year intervals [105]. On each control visit, a venous blood sample was collected, and a patient underwent ECG examination. A total of 6894 patients turned up on a second visit, of which 5452 had available measurements of micronutrients concentration including Se [106]. A mean follow-up was 6.2 years, during which 136 participants developed AF. Of 3900 non-smoking participants with Se deficiency established as <70 μg/L, the risk of developing AF was 68% higher than in the general population (95% CI 1.09 to 2.59, *p* = 0.018). In a group above the age of 60, this association remained significant also among smoking participants. Lowered Se levels were also encountered in patients with newly diagnosed AF compared to healthy subjects in study by Ardahanli and Ozkan [107]. However, this association was statistically significant only in men but not in women. This was attributed to differences in nutrition and concurring risk factors. Bazargani et al. observed that patients with AF have not only lowered the level of Se but also zinc, while the level of copper decreased [108].

Measurements of Se were also conducted during episodes of paroxysmal AF and were evaluated 24 h and 28 days after restoration of sinus rhythm [109]. Statistically significant lowered values were obtained only during arrhythmic episodes, remaining unaltered at further evaluations. All these findings are not surprising given the fact that fibrosis, inflammatory mediators, and oxidative stress are predisposing factors to arrhythmias. Each 10% increase in redox potential of glutathione is associated with a 40% risk of an AF incident [110].

There is still a lack of proper studies covering isolated use of Se in treatment of AF. Mirhoseini et al. analyzed data from 4699 critically ill patients admitted to the intensive care unit [111]. The group, which received a mix of antioxidants (i.e., vitamin C, vitamin E and Se), did not display better outcomes in terms of incidence reduction in atrial arrhythmias compared to controls in the first two weeks of hospitalization (3.02% vs. 3.31%, *p* = 0.62). However, the antioxidant group also did not display decreased mortality; it had longer expected survival time. Vitamins C and E were shown to reduce risk of AF recurrence after successful cardioversion but also to treat ventricular arrhythmias and arrhythmias linked with long-QT interval. Therefore, further trials are necessary in order to pinpoint effects of Se use in arrhythmic diseases besides AF [112].

In addition to its idiopathic etiology, AF is frequently recognized as a complication after both cardiac, thoracic, and to a lesser extent, other types of surgeries. Most commonly, it occurs within 48 h after the procedure and typically is a transient phenomenon. Its incidence was estimated from 10% up to 60% of cardiac surgeries, depending on the type of procedure with the highest risk related to valve replacement. The risk progressively increases in line with age [113]. Its pathomechanism is not yet completely understood. It is known that both acute surgical factors and progressive remodeling contribute to its development. Excessive oxidative stress and altered mechanisms of its prevention are considered to be two of the major factors [114]. Among patients who underwent intermediate risk coronary artery surgery, it was shown that low preoperative Se is associated with postoperative AF [72]. On the other hand, Kyaruzi et al. found no statistically significant difference in concentration level of Se between patients with and without AF [115].

### 2.5. Cardiac Surgeries

Se is recognized as a factor affecting outcomes of cardiac surgeries. Koszta et al. observed that patients who died following cardiac surgery had lower levels of blood Se compared to survivors (mean 102.2 μg/L vs. 111.1 μg/L, *p* = 0.047) [116]. It was considered a minor risk factor of postoperative mortality. It is yet unclear whether Se supplementation would be beneficial in terms of survival.

In a prospective study by Stoppe et al., sixty patients were evaluated on concentration of Se, copper, and zinc before and after cardiac surgeries with the use of a cardiopulmonary bypass [117]. Se concentration measured one hour after admission to the intensive care unit showed that it is an independent predictor of postoperative multiorgan failure occurrence within the next 48–72 h. Among patients with no organ failure, its level was 72.2 μg/L on average, while in patients with multiorgan failure, it was 61.5 μg/L. Se concentration also correlated with length of stay and perioperative inflammation. A similar pattern was also reported by Zhou et al., but there was no difference in concentration of Se between groups with AF and those who were in sinus rhythm [118]. The levels of Se after open-heart surgery also varied with regard to duration of a procedure, length of intensive care unit stay, and duration of cardiopulmonary bypass [119]. Similarly, Stevanovic et al. found low levels of Se as a predictive factor for developing complications and negative correlation to myocardial damage monitored by levels of CK-MB (r = −0.571, *p* < 0.001) [120].

There were attempts to determine whether Se perioperative administration would be beneficial in reducing post-surgical adverse effects. However, no significant differences were observed in the reduction in mortality, acute kidney injury, length of hospitalization and intensive care unit stay, CK-MB, and troponin I levels compared to placebo groups [121]. SUSTAIN CSX trial assessed the ability of selenite treatment in reducing postoperative organ dysfunction and mortality in 1416 cardiac surgery patients. Their study did not confirm the benefits of perioperative Se supplementation in patients with high cardiac risk [122]. However, Schmidt et al. managed to obtain a reduction in postoperative vasoactive support with a high dose of sodium selenite [123]. The impact of Se supplementation on the metabolic status of patients undergoing coronary artery bypass grafting was examined by Kamali et al. [124]. A four-week intake of Se at a dose of 200 μg/day resulted in an elevation of HDL cholesterol levels and glutathione and a reduction in fasting plasma glucose, insulin, malondialdehyde, and high sensitivity CRP [124].

Although there are available data from clinical trials assessing the effectiveness of single Se supplementation, its usage in combination with other agents still requires further evaluation [121].

## 3. The Influence of Selenium in the Environment and Selenium-Enriched Diet on Human Health

**Figure 2:**
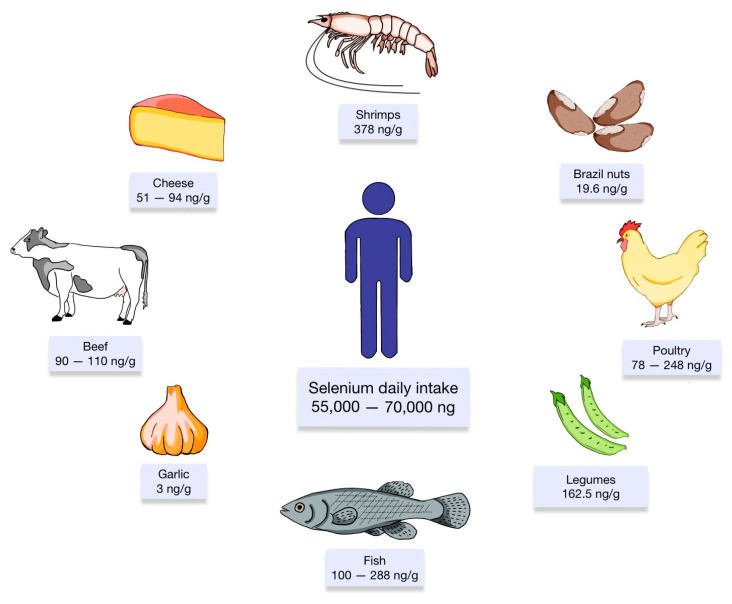
Food products rich in Se. Based on the studies of Juszczak et al. [132] and Pappa et al. [133].

Adequate levels of Se ensure homeostasis not only in cardiovascular systems, but also endocrine, reproductive, and nervous systems [125,126]. In food, Se is typically present in trace amounts, measuring ≤ 1 mg/kg, with some exceptions such as Brazilian nuts, which can contain even 20 mg Se per kg [127]. The content of Se in various foods is presented in Figure 2. The recommended Se intake depends on age, sex, and physiological status. For adults, the recommended daily intake ranges between 55 and 70 µg/day [128,129]. Pregnant women should aim for 65 µg/day, while breastfeeding mothers require slightly more Se, approximately 75 µg/day [130]. In healthy individuals, the plasma Se concentrations around 100 µg/L recorded the highest selenoprotein activity [131]. It is essential what the maximum tolerable intake is, because of side effects that may occur [126].

Organic forms of Se are typically encountered as sulfur amino acid analogs, notably selenomethionine and SeC, while inorganic Se exists in the form of Se salts, selenate (SeO−24) and selenite (SeO−23) [134]. Both organic and inorganic forms can serve as valuable dietary sources; however, the inorganic forms appear to be more toxic than the organic ones [135]. The inorganic forms of Se are mostly found in seafood, legumes, and dry fruits [127]. They are mostly used in biofortification and in supplements. The selenate, emerging as the most prevalent one, is detected primarily in water [136], but can also be found in wheat straw [137]. The organic variants dominate in animal products, such as beef and poultry. Apart from that, selenomethionine can also be found in vegetables, grains, legumes, nuts, and yeast, along with being present in dietary supplements [138]. SeC is present in poultry, beef, and dairy products, while selenium-methylselenocysteine is primarily detected in Allium vegetables like garlic, onion, broccoli, and leeks [139,140]. The primary factor influencing Se concentration in plant-based foods, and to some extent in animal-derived ones, is the availability of Se in the environment, especially in the soil [141]. Se is present in both soil and water, where it can be mobilized and incorporated into the food chain through plant roots or aquatic organisms. This phenomenon raises concerns about potential long-term effects on human health [142,143].

**Figure 3:**
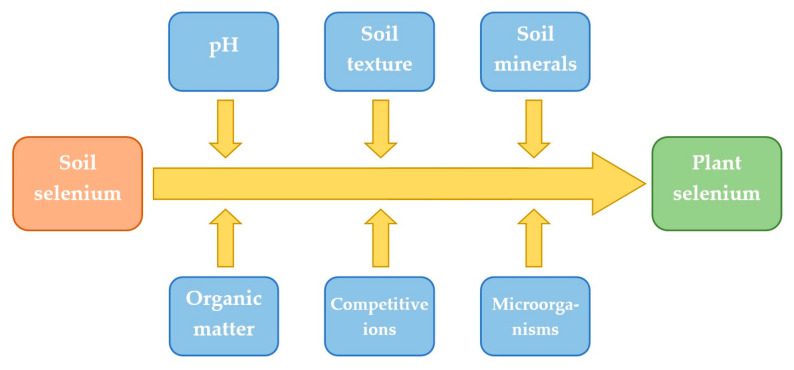
Soil properties affecting the bioavailability of selenium to plants [148].

Depending on the region of crop cultivation and animal husbandry, the Se content in foods may vary [144]. This variability necessitates careful consideration when devising strategies for Se supplementation. However, it is worth noting that the Se concentration in water and soil is not the sole factor influencing its uptake by plants. Soil Se mobility processes and other factors such as soil pH or organic matter content also significantly affect its availability to plants (Figure 3). Plants uptake Se in organic forms, such as selenate and selenite, to a much greater extent than in inorganic forms, and some plant species have the ability to accumulate this microelement in high concentrations, making them Se hyperaccumulators [145]. The environmental role of Se in the atmosphere and its effect on human health is an understudied topic. Studies suggest that in general, atmospheric background levels of Se are low in urban areas [145]. Further research is necessary to assess in greater detail Se levels in rural areas and its role in agriculture. This is associated with selenium’s potentially toxic effects on natural ecosystems due to its bioaccumulation potential. Se found in water and soil can be mobilized to enter the food chain, which is coupled with concerns about its long-term environmental effects on humans and ecosystems [145]. Most toxicity levels are based on absorption in the aquatic environment, which is much lower in natural populations than absorption in the food chain [146]. According to Tan et al., 40% of Se emissions in the atmosphere and aquatic environment come from human industrial activities such as mining, refineries, agriculture, and coal-fired power plants [147]. Overall, the limited existing research indicates that monitoring of environmental Se levels should be considered. This would further aid in developing appropriate management strategies to minimize the risk of toxicity to humans.

The pharmacokinetics of Se therapy and the general health impact depend not only on the chemical form of this trace element but also its bioavailability and the mode of administration [149]. It is important to underline that organic Se, particularly SeMet, is characterized by higher bioavailability [128,150] and offers more benefits than its inorganic counterpart within the context of oral supplementation and a well-rounded diet [151]. The most efficacious parenteral forms of Se supplementation are inorganic selenocompounds, and its organic forms often are not available for intravenous use [152].

Excessive Se intake leads to a change in the mechanism of action of this trace element on tissues; it becomes a pro-oxidant and, as a result, causes oxidative harm to cells and tissues and occurring side effects [126,153]. However, the toxic one-time dose is not precisely known [154]. The lethal one-time dose seems to oscillate around 4000 μg [154]. Acute toxicity manifests with diarrhea, fatigue, stomach and joint pain, respiratory issues, nausea, and hair loss [155]. 

Considering the chronic delivery of Se to the body, the lowest observed adverse effect levels of 3–4 µg Se/kg body weight/day in humans are close to normal consumption levels, ranging from 0.15 to 4 µg/kg body weight/day. Consequently, Se can be viewed as a trace element with a narrow concentration range in the human body, spanning from deficiency to optimal physiological levels and toxicity [149]. The chronic ingestion of excessive Se leads to selenosis. This condition manifests with a range of symptoms including skin rash, hair loss, tooth discoloration, halitosis reminiscent of garlic, gastrointestinal disturbances, numbness, paralysis, and hemiplegia [156,157].

Se deficiency, which affects about one billion people in the world, develops due to its insufficient consumption. This factor mainly depends on the geographical area and correlates with the low content of this microelement in the soil. The increased risk of mortality rates among patients with HF, development of type 2 diabetes mellitus, and heightened incidence of prostate cancer have been associated with Se deficiency [158,159,160]. This underscores the importance of maintaining Se intake within safe limits to avoid detrimental health consequences. Also, the diminished Se levels in the body are implicated in compromised fetal development, male infertility, and heightened susceptibility to asthma [130,161,162]. These effects stem from the attenuation of antioxidant defenses and the reduction in activity of glutathione peroxidase [126].

Furthermore, Se deficiency combined with coxsackievirus B3 infection leads to Keshan disease (KD), an endemic cardiomyopathy, reported in China [163]. KD was first identified in Keshan County in 1935, and due to its occurrence, the Chinese government introduced a Se supplementation program in the 1970s. Prior to the implementation of the program, adults residing in regions afflicted by KD demonstrated about 11 µg Se intakes a day [156]. It has been established that Se intakes of at least 20 mcg/day protect adults from KD [164]. The pathogenesis of KD is not yet fully understood, but the presence of infection, which leads to oxidative stress, is also an important risk factor for its development. This hypothesis is supported by the study by Pei et al., which demonstrated that lower activity of selenoproteins is a component of the disease in patients with KD [165]. It seems that the most crucial etiologic factors considered are deficiency of Se in diet and environment, above others considered, including toxins, viral infections, and lack of vitamins or minerals. In the study by Zhang et al., performed on animal models, it was observed that low Se levels affect PINK1/Parkin-mediated mitochondrial autophagy pathway, which exacerbate damage to the myocardium in KD [166]. Yet, the main evidence in Se involvement are benefits in patients receiving sodium selenite supplementation in endemic areas [138]. The clinical presentations observed in patients with KD includes congestive HF, cardiogenic shock, and arrhythmias [163]. In the imaging exam, dilatation of the heart cavities is observed, as well as numerous necrotic foci with various degrees of fibrosis [167].

### Selenium Supplementation

Selenoproteins play a crucial role in preventing oxidative modification, thereby reducing inflammation and inhibiting platelet aggregation (Table 1). Due to these functions, it has been suggested that Se supplementation could reduce the risk of CVD and its risk of mortality. The meta-analysis of 16 prospective observational studies revealed a significant beneficial range of Se concentration between 55 and 145 μg/L, which was related to a lower risk of CVD development [168]. However, none of the nine randomized controlled trials indicated any overall effect of Se on CVD risk.

The necessity of adding Se to the antioxidant mix is also confirmed by another meta-analysis that analyzed 43 randomized controlled trials [101]. This study revealed a reduced risk for all-cause mortality when Se was part of the antioxidant mix (RR: 0.90; 95% CI: 0.82, 0.98; *p* = 0.02). Conversely, an antioxidant mix without Se showed indications of increased risk (RR: 1.09; 95% CI: 1.04, 1.13; *p* = 0.0002; I2 = 0%) for all-cause mortality. This research highlights the necessity of integrating Se into any antioxidant supplement mix to optimize its efficacy and mitigate potential CVD or all-cause mortality risk.

Another meta-analysis of 16 randomized controlled trials, involving 43,998 participants, studied the effect of Se supplementation on CHD [54]. The results of this study showed significant effects of Se supplementation on lowering serum CRP and increasing GSH-PX. However, there was no statistically significant association between Se supplementation and CHD mortality or improving their lipid profiles. Also, in another meta-analysis, to which patients were subjected to various micronutrient supplementation, Se had a neutral effect on CVD; its supplementation showed no therapeutic effects on lipid profile, blood pressure, myocardial infarction, stroke, or coronary heart disease [169].

Overall, the current clinical evidence does not support the routine use of Se supplements for preventing or treating heart disease, especially among patients who already maintain adequate serum Se levels. Further clinical trials are necessary to evaluate the contributions of dietary Se intake and supplementation in patients with CVD.

## 4. Conclusions

In conclusion, the current literature suggests that Se plays a crucial role in myocardial tissue homeostasis and in the progression of CVD. It has been shown that low levels of Se correlate with the risk of developing AF, both pre- and post-operatively. Attempts at supplementation have not yielded statistically significant results, both in reducing AF incidence and CHD mortality. However, these results demonstrated notable effects on GPx activity and serum CRP levels, offering potential benefits in managing CHD risk factors. Supplementation of this micronutrient appears promising in the prevention and treatment of HF. Despite the clear links that Se has to CVD development, further research is still required to assess the clinical power of these findings.

## Data Availability

The data used in this article are sourced from materials mentioned in the References section.
